# Preparation and antibacterial properties of titanium-doped ZnO from different zinc salts

**DOI:** 10.1186/1556-276X-9-98

**Published:** 2014-02-27

**Authors:** Tong Sun, Han Hao, Wen-ting Hao, Shu-min Yi, Xue-peng Li, Jian-rong Li

**Affiliations:** 1 College of Chemistry, Chemical Engineering and Food Safety, Bohai University, Jinzhou 121013, People's Republic of China; 2 Food Safety Key Laboratory of Liaoning Province, Bohai University, Jinzhou 121013, People's Republic of China

**Keywords:** Antibacterial, Microstructure, Alcohothermal method, Zinc oxide, Titanium-doped

## Abstract

To research the relationship of micro-structures and antibacterial properties of the titanium-doped ZnO powders and probe their antibacterial mechanism, titanium-doped ZnO powders with different shapes and sizes were prepared from different zinc salts by alcohothermal method. The ZnO powders were characterized by X-ray powder diffraction (XRD), Fourier transform infrared spectroscopy (FT-IR), ultraviolet-visible spectroscopy (UV-vis), scanning electron microscopy (SEM), transmission electron microscopy (TEM), and selected area electron diffraction (SAED), and the antibacterial activities of titanium-doped ZnO powders on *Escherichia coli* and *Staphylococcus aureus* were evaluated. Furthermore, the tested strains were characterized by SEM, and the electrical conductance variation trend of the bacterial suspension was characterized. The results indicate that the morphologies of the powders are different due to preparation from different zinc salts. The XRD results manifest that the samples synthesized from zinc acetate, zinc nitrate, and zinc chloride are zincite ZnO, and the sample synthesized from zinc sulfate is the mixture of ZnO, ZnTiO_3_, and ZnSO_4_ · 3Zn (OH)_2_ crystal. UV-vis spectra show that the absorption edges of the titanium-doped ZnO powders are red shifted to more than 400 nm which are prepared from zinc acetate, zinc nitrate, and zinc chloride. The antibacterial activity of titanium-doped ZnO powders synthesized from zinc chloride is optimal, and its minimum inhibitory concentration (MIC) and minimum bactericidal concentration (MBC) are lower than 0.25 g L^−1^. Likewise, when the bacteria are treated by ZnO powders synthesized from zinc chloride, the bacterial cells are damaged most seriously, and the electrical conductance increment of bacterial suspension is slightly high. It can be inferred that the antibacterial properties of the titanium-doped ZnO powders are relevant to the microstructure, particle size, and the crystal. The powders can damage the cell walls; thus, the electrolyte is leaked from cells.

## Background

Antibacterial agents are applied to many fields, such as food [1,2], care [3], packaging [4], synthetic textiles [5], environmental [6], and so on. Chemical synthesis antibacterial agent is divided into two categories: organic and inorganic antibacterial agent [7-9]. Organic antibacterial agent has many disadvantages, including the toxicity hazard to the human body and instability in high temperature and pressure [10]. By comparison, inorganic antibacterial agent has the properties of heat resistance, long life, and chemical stability [11]. Nowadays, metallic simple substances and their compounds are used widely in antimicrobial application research, such as Ag [12-16], Fe_2_O_3_[17], TiO_2_[18], CuO [19,20], MgO [21], Mg (OH)_2_[22], and ZnO [23,24]. Among metal oxide antibacterial agents, ZnO has aroused concern due to its good antibacterial activities on a broad spectrum of bacteria [24-26]. The antibacterial properties of ZnO have been studied broadly with pathogenic and nonpathogenic bacteria such as *Staphylococcus aureus*, *Escherichia coli*, *Klebsiella pneumoniae*, *Pseudomonas*, etc. [26,27]. Zinc oxide is an interesting material due to its extensive applications in various areas, such as antibacterial, optical, piezoelectric, magnetic, and gas sensing properties [24,26-31]. Therefore, many of the synthetic approaches such as sol-gel method [32], co-precipitation [31], hydrothermal method [33], microwave synthesis [23,26], and thermal evaporation method [34] have been used for the preparation of ZnO powders. Hydrothermal method is an important technology in synthetic material. Using this method, the crystal grain can develop completely and the particle size is uniform.

In this work, in order to research the influence of the microstructure and crystal on the antibacterial properties of titanium-doped ZnO powders, the powders were synthesized by alcohothermal method from different zinc salts, and the antibacterial activities against *E. coli* and *S. aureus* were evaluated. Moreover, the antibacterial mechanism of titanium-doped ZnO powders was deduced.

## Materials and methods

### Materials

The reagents (e.g., two hydrated zinc acetate, zinc vitriol, zinc nitrate, zinc chloride, lithium hydroxide monohydrate, absolute ethyl alcohol, tetrabutyl titanate, glutaraldehyde, disodium hydrogen phosphate 12-hydrate, monopotassium phosphate) used in this study were analytically pure chemicals. Biological reagents (e.g., nutrient broth, nutrient agar medium) were used as received. De-ionized water and *aquae sterilisata* with conductivity lower than 0.5 μS/cm were used to prepare all the solutions. *E. coli* (ATCC44104) and *S. aureus* (CMCC26001) bacterial strains were obtained from Beijing Assay Institute of Biological Products. Phosphate-buffered saline (PBS; pH = 7.4) was prepared with disodium hydrogen phosphate 12-hydrate and monopotassium phosphate.

### Synthesis of titanium-doped ZnO powders

Under magnetic stirring condition, 0.1 mol/L zinc salts and 0.14 mol/L lithium hydroxide alcoholic solution were prepared. Meanwhile, 0.01 mol/L tetrabutyl titanate alcoholic solution was prepared. Then, 80 mL of lithium hydroxide alcoholic solution was dropwise added to 160 mL zinc salt alcoholic solution during the stirring process. After that, 80 mL of tetrabutyl titanate alcoholic solution was added to it drop by drop. Subsequently, 8 mL of deionized water was added into the mixed solution, and then the mixed solution was treated by ultrasound for 1 h. The mixed solution was shifted into the hydrothermal reactors with 70% filling, and then the reactors were sealed and heated for 24 h at 140°C. After the reactors were cooled naturally to room temperature, the precipitates were collected and washed several times using distilled water and then were dried at 40°C. After grinding, the titanium-doped ZnO powders were prepared.

### Evaluation of antibacterial activity

Bacterial strains (*E. coli* and *S. aureus*) were cultured overnight in nutrient broth medium at 37°C before being used. The strains were diluted to 10^5^ to 10^6^ colony forming units (CFUs) per milliliter with PBS. Twenty milliliters of dilute bacterial suspension was taken in each of the iodine number flask, respectively. The powders of 0.25 to 2.5 g/L were added into each flask. The bacterial suspension without powders was used as positive control. All the iodine number flasks were put on a shaker bed at 150 rpm and incubated at 37°C for 24 h. Both the treated and control bacterial suspensions were diluted by a series of twofold dilutions in PBS solution. The dilute solutions with appropriate dilution ratio were then plated on nutrient agar plates to assay the colony forming ability. Plates were incubated at 37°C for 48 h, and the colonies were counted. All experiments were performed in triplicate, and the averages were obtained.

### Characterization of titanium-doped ZnO powders

The crystalline phases of the powders were characterized by X-ray powder diffraction (XRD) using D/MAX-RB X-ray diffractometer (Rigaku, Tokyo, Japan) with Cu K radiation in the 2*θ* range of 10° to 70° at a scan rate of 8°/min. Fourier transform infrared spectra (FT-IR) of the powders were characterized using Scimitar 2000 Near FT-IR spectrometer (Thermo Electron, Madison, WI, USA), and the spectra were recorded in the range of 4,000 to 400 cm^−1^. The UV-visible diffuse reflectance spectra of the powders were recorded with a model Shimadzu UV2550 spectrophotometer (Shimadzu, Nakagyo-ku, Kyoto, Japan). The morphologies of the powders were examined by field emission scanning electron microscopy (FESEM; S-4800, Hitachi, Ltd., Chiyoda, Tokyo, Japan) and field emission transmission electron microscopy (FETEM; JEM-2100 F, JEOL Ltd., Akishima, Tokyo, Japan). Meanwhile, the crystalline characters of the powders were examined.

### Characterization of cells' morphology

Fresh bacterial culture was treated with titanium-doped ZnO powders at 37°C for 18 h, and then the bacterial suspension of control and treatment were fixed with 2.5% (*v/v*) glutaraldehyde for 2.5 h. After being centrifuged at 2,500 rpm for 5 min, the liquid supernatant of bacterial suspension was discarded. After being added *aquae sterilisata* and centrifuged for three times, a drop of bacterial suspension was placed on the cover slip and dried under vacuum. The bacterial cells' morphologies were examined using FESEM (S-4800, Hitachi).

### Measurement of the electrical conductance of bacterial suspension

The *E. coli* and *S. aureus* in logarithmic phase were washed with *aquae sterilisata* for three times, and then the concentration of the bacterial suspension was adjusted to 10^7^ CFU/mL. One hundred milligrams of titanium-doped ZnO powders were added to 50 mL 10^7^ CFU/mL of bacterial suspension. The bacterial suspension without powders was used as control. The electrical conductance of the bacterial suspension was measured with 10-min interval. All experiments were performed for three times, and averages were obtained.

## Results and discussion

### XRD characterization of titanium-doped ZnO powders

**Figure 1:**
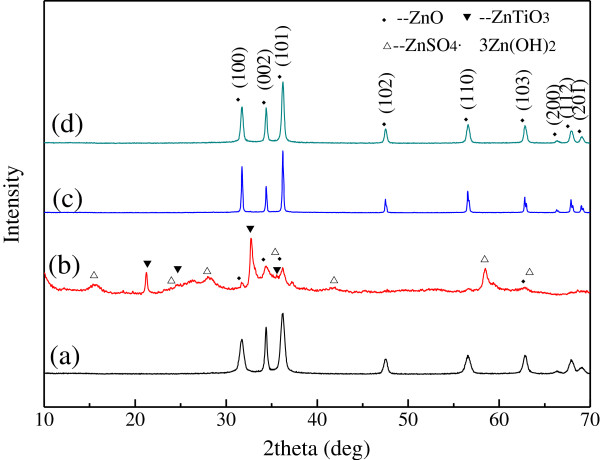
**XRD patterns of the titanium-doped ZnO powders synthesized from different zinc salts.** (a) Zinc acetate, (b) zinc sulfate, (c) zinc nitrate, and (d) zinc chloride.

Figure 1 shows the crystalline peaks of titanium-doped ZnO powders synthesized by alcohothermal method. The diffraction peaks at 2*θ* values of 31.7°, 34.4°, 36.2°, 47.5°, 56.5°, 62.8°, 66.3°, 67.9°, and 69.0° correspond to the (100), (002), (101), (102), (110), (103), (200), (112), and (201) planes of ZnO, respectively, and can readily be indexed to the hexagonal zincite (PDF#36-1451). All the samples have the same diffraction peaks except those synthesized from zinc sulfate. The powders synthesized from zinc sulfate have diffraction peaks of ZnTiO_3_ and ZnSO_4_ · 3Zn (OH)_2_. In all XRD patterns, none of these peaks for titanium compound are observed. This may be ascribed to the fact that titanium molecules occupy some sites originally accessible to zinc molecules or exist as amorphous. In addition, the peaks of titanium-doped ZnO powders synthesized from zinc nitrate and zinc chloride are sharper than others, and the half peak width is narrower. This suggests that these two samples' crystallinity is better and the size is larger.

### FT-IR spectra of titanium-doped ZnO powders

**Figure 2:**
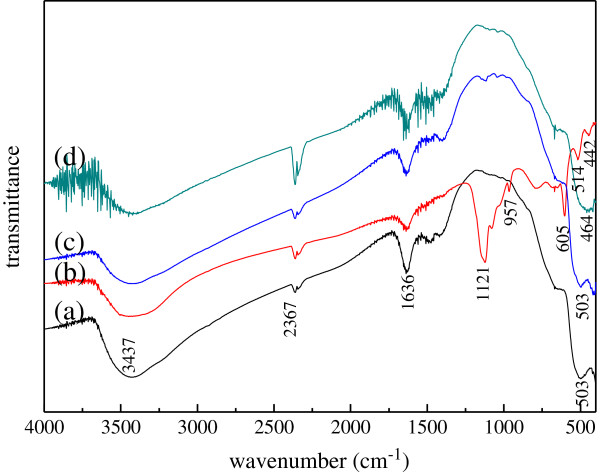
**FT-IR spectra of the titanium-doped ZnO powders synthesized from different zinc salts.** (a) Zinc acetate, (b) zinc sulfate, (c) zinc nitrate, and (d) zinc chloride.

Figure 2 shows the FT-IR spectra of the titanium-doped ZnO powders, which were acquired in the range of 4,000 to 400 cm^−1^. All of the spectra exhibit a strong absorption peak at 3,500 to 3,300 cm^−1^ for stretching vibration of non-chemical bond association OH groups and at 1,636 cm^−1^ for H-O-H bending vibrations. It indicates that the pore water or adsorbed water is in the powders [35]. The peaks at 2,367 cm^−1^ are attributed to the presence of carbon dioxide. The peaks at 514 to 442 cm^−1^ are for Zn-O or Ti-O, and the peaks at 605 cm^−1^ are for Zn-OH or Ti-OH [10,36]. Meanwhile, the peak at 1,211 cm^−1^ corresponds to characteristic absorption peaks of SO_4_^2−^. Figure 2(a, c, d) shows that there are Zn-O and Ti-O bands in the samples, and Figure 2(b) shows that there are SO_4_^2−^, Zn-OH or Ti-OH, Zn-O, and Ti-O bands in the titanium-doped ZnO powders synthesized from zinc sulfate. The above results are in accordance with the XRD results.

### UV-visible spectra of titanium-doped ZnO powders

**Figure 3:**
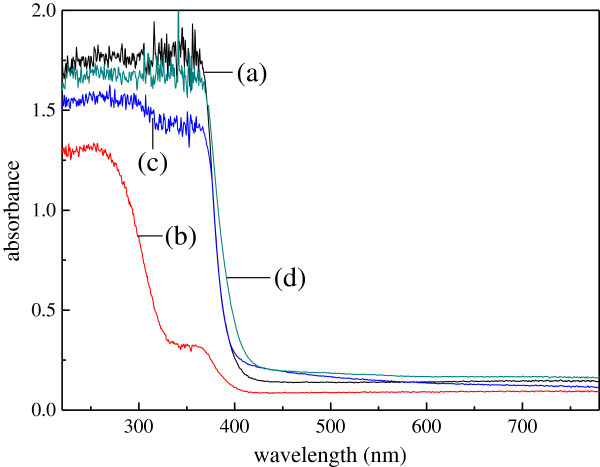
**UV-visible spectra of the titanium-doped ZnO powders synthesized from different zinc salts.** (a) Zinc acetate, (b) zinc sulfate, (c) zinc nitrate, and (d) zinc chloride.

Figure 3 shows the UV-visible absorption spectra of the titanium-doped ZnO powders. From Figure 3(a, c, d), it can be seen that the absorption edges of the titanium-doped ZnO powders are more than 400 nm, which were synthesized from zinc acetate, zinc nitrate, and zinc chloride. However, Figure 3(b) shows that the absorption edge wavelength of the powders is less than 400 nm. Because the absorption edge of the zincite ZnO is 387 nm [28], it is demonstrated that the absorption edge shift of the powders are due to the particle size and crystal structure. When the titanium-doped ZnO powders are synthesized from zinc acetate, the particle size is smaller than the others, and their quantum size effect is enhanced. Likewise, titanium gets into the crystal lattice of the zinc oxide, and the crystal lattice is destroyed; thus, the band gap is decreased. For these reason, red shift effect is caused. The absorption edge wavelength of the titanium-doped ZnO powders synthesized from zinc acetate and zinc nitrate is equal, but the particle size of the powders synthesized from zinc nitrate is larger than the powders synthesized from zinc acetate. The reason might be that the doping effect of the powders synthesized from zinc nitrate is better than the powders synthesized from zinc acetate. In addition, the absorption edge wavelength of the powders synthesized from zinc chloride is longer than the others. This is due to the particles which are smaller than the others. In addition, using zinc sulfate as zinc salt, the absorption edge of the samples is less than the other. It may be for two reasons. The first is there are ZnO, ZnTiO_3_, and ZnSO_4_ · 3Zn (OH)_2_ crystals, and the composite semiconductors cannot make the band gap decrease. The second is their poor quantum size effect due to irregular powders.

### SEM characterization of titanium-doped ZnO powders

**Figure 4:**
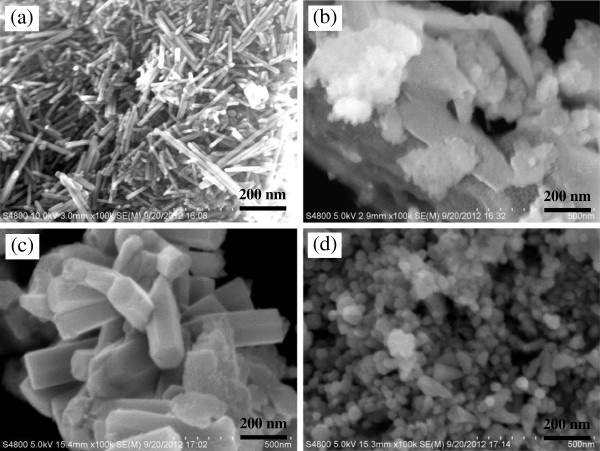
**SEM images of the titanium-doped ZnO powders synthesized from different zinc salts. (a)** Zinc acetate, **(b)** zinc sulfate, **(c)** zinc nitrate, and **(d)** zinc chloride.

Figure 4 shows the scanning electron microscope (SEM) images of titanium-doped ZnO powders. The morphologies of the samples are different obviously with each other. This suggests that the morphologies of powders are deeply affected by the raw material. Figure 4a shows that the powders synthesized from zinc acetate are rod shape with a diameter about 20 nm and varying lengths. As shown in Figure 1(a), when the zinc salt is zinc acetate, the diffraction peak intensity of (002) crystal face is stronger than PDF#36-1451; it means that the prior growth direction of zinc oxide crystal is [0001]. For this reason, the powders are rod shape as shown in Figure 4a. When the raw material is zinc sulfate, the powders are irregular including aggregate particles and big sheets (Figure 4b). The samples prepared from zinc nitrate are six prismatic with a diameter about 120 nm (Figure 4c). As shown in Figure 1, the XRD diffraction peaks of the samples synthesized from zinc nitrate are attributed with PDF#36-1451, and the diffraction peaks' height ratio of (100), (002), and (101) crystal face is the same as PDF#36-1451. Therefore, the samples are shown in perfect six prismatic of hexagonal zincite. Figure 4d shows that the powders prepared from zinc chloride are spherical and tooth shape with a diameter around 40 to 70 nm. Figure 1 shows that the diffraction peaks of (100) and (002) crystal face are stronger than that of PDF#36-1451. So, the zinc oxide crystals are preferentially grown along the direction of [1000] and [0001], and the powders mostly become spherical and tooth shape.

### TEM characterization of titanium-doped ZnO powders

**Figure 5:**
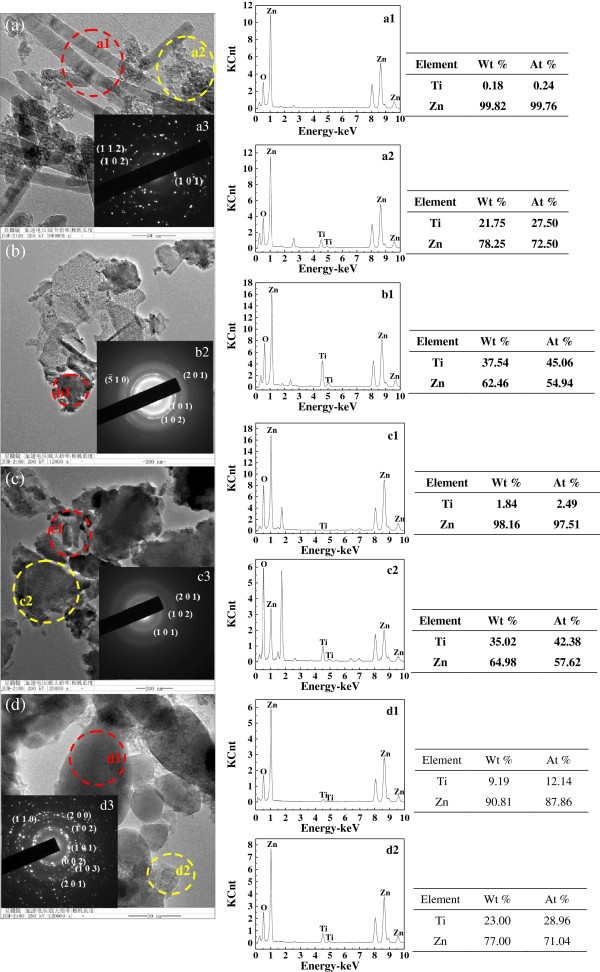
**TEM images, SAED, and EDS of the titanium-doped ZnO powders synthesized from different zinc salts.** TEM images: **(a)** zinc acetate, **(b)** zinc sulfate, **(c)** zinc nitrate, and **(d)** zinc chloride. EDS: a1, a2 - zinc acetate; b1 - zinc sulfate; c1, c2 - zinc nitrate; d1, d2 - zinc chloride. SAED: a3 - zinc acetate; b2 - zinc sulfate; c3 - zinc nitrate; d3 - zinc chloride.

As shown in Figure 5, the structural morphologies of the titanium-doped ZnO powders were further characterized by transmission electron microscope (TEM), and the composition were characterized by selected area electron diffraction (SAED) patterns and energy-dispersive spectrometry (EDS) spectrums. Compared with the SEM image, the TEM image shows that the samples synthesized from zinc acetate also contain small nanoparticles besides nanorods (Figure 5a). Figure 5b reveals that the sheets synthesized from zinc sulfate are made up of small nanoparticles. Apart from six prismatic particles shown in the SEM image, the samples prepared from zinc nitrate also contain sheets (Figure 5c). When the raw material is zinc chloride, the samples also contain small nanoparticles besides spherical and dentiform particles (Figure 5d).

The EDS spectrums (Figure 5(a1, a2)) of the samples synthesized from zinc acetate show that titanium is almost undetected in the rods, yet the fine particles next to the rods contain a certain amount of titanium. It indicates that the titanium is not doped in the ZnO and there is amorphous substance in the samples. This is why the titanium is not detected in the XRD. Figure 5(b1) shows that a large number of titanium is in the agglomerate substance of the samples synthesized from zinc sulfate. When the samples are prepared from zinc nitrate, EDS results (Figure 5(c1, c2)) show that the sheets contain more titanium than the rods. Similarly, (Figure 5(d1, d2)) shows that the fine particles contain more titanium than the formed particles which is synthesized from zinc chloride. Meanwhile, the atomic percentage content of titanium in the tooth shape particles is 12.14%; it is almost consistent with the experimental process in which the molar ratio of titanium and zinc is 1 to 10. It manifests that titanium is almost utterly doped in the ZnO.

The crystalline characters of the samples are checked by selected area electron diffraction. Figure 5(a3) shows that samples synthesized from zinc acetate have certain crystalline state, and the crystalline grain size is slightly larger. The (101), (102), and (112) crystal faces are detected. This is consistent with the XRD. When the raw material is zinc sulfate, the diffraction pattern displays the ($\bar{5}$ 10) lattice plane of Zn (SO_4_)_2_ · 3Zn (OH)_2_ and (101), (102), and (201) lattice planes of ZnO (Figure 5(b2)). The result is consistent with the XRD. When the raw material is zinc nitrate, (101), (102), and (201) crystal planes of ZnO are detected, and the diffraction rings are obscure (Figure 5(c3)). It demonstrates that the samples are composed of amorphous and crystalline forms. The SAED pattern of the samples prepared from zinc chloride displays the (002), (101), (102), (110), (103), (200), and (201) crystal planes of ZnO (Figure 5(d3)). It indicates that the samples are hexagonal phase. Besides, there are some scattered bright spots in the diffraction pattern. It demonstrates that the grain size is slightly larger.

### Antibacterial properties of titanium-doped ZnO powders

Tables 1 and 2 both show that the antibacterial activities of titanium-doped ZnO powders synthesized from different zinc salts is different. The antibacterial activities of the powders are optimal, which is prepared from zinc chloride, and their minimum inhibitory concentration (MIC) and minimum bactericidal concentration (MBC) are lower than 0.25 g L^−1^. Moreover, the antibacterial properties of the powders synthesized from zinc nitrate are slightly poorer than that of zinc chloride and are better than that of zinc acetate and zinc sulfate. Meanwhile, the antibacterial activities of the powders against *E. coli* are better than *S. aureus*.

**Table 1:** **Colony count of *E. coli *** **after antibacterial activities by titanium-doped ZnO powders**

**Zinc salt**	**Powder concentration (g/L)**							
	**0**	**0.25**	**0.5**	**0.75**	**1.0**	**1.5**	**2.0**	**2.5**
Zn (Ac)_2_	1.25 × 10^8^	2.1 × 10^7^	1.95 × 10^7^	1.75 × 10^7^	1.2 × 10^7^	3.85 × 10^6^	2.9 × 10^3^	1.65 × 10^3^
ZnSO_4_		1.1 × 10^7^	9.75 × 10^6^	5.3 × 10^6^	2.95 × 10^5^	5.6 × 10^4^	1.6 × 10^4^	7.65 × 10^3^
Zn (NO_3_)_2_		2.15 × 10^7^	1.9 × 10^7^	1.65 × 10^7^	1.6 × 10^7^	3.35 × 10^5^	2.8 × 10^3^	0
ZnCl_2_		3.05 × 10^4^	6.55 × 10^3^	3.9 × 10^3^	2.5 × 10^3^	2.3 × 10^3^	2.0 × 10^3^	0

The initial bacterial colony count is 8.75 × 10^5^ CFU/mL.

**Table 2:** **Colony count of *S. aureus *** **after antibacterial activities by titanium-doped ZnO powders**

**Zinc salt**	**Powder concentration (g/L)**							
	**0**	**0.25**	**0.5**	**0.75**	**1.0**	**1.5**	**2.0**	**2.5**
Zn (Ac)_2_	1.95 × 10^8^	5.25 × 10^7^	5.2 × 10^7^	4.0 × 10^7^	3.4 × 10^7^	3.0 × 10^7^	4.15 × 10^5^	2.1 × 10^3^
ZnSO_4_		8.85 × 10^7^	8.3 × 10^7^	7.55 × 10^7^	4.35 × 10^7^	4.0 × 10^7^	6.25 × 10^6^	2.0 × 10^5^
Zn (NO_3_)_2_		9.65 × 10^7^	9.15 × 10^7^	8.9 × 10^7^	8.3 × 10^7^	1.01 × 10^7^	2.6 × 10^5^	6.0 × 10^2^
ZnCl_2_		7.35 × 10^4^	5.6 × 10^4^	2.0 × 10^4^	3.5 × 10^3^	1.9 × 10^3^	1.7 × 10^2^	34

The initial bacterial colony count is 9.9 × 10^5^ CFU/mL.

### SEM characterization of *E. coli* and *S. aureus* cells

**Figure 6:**
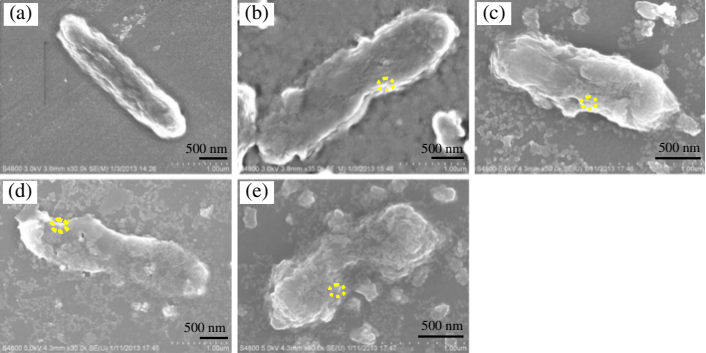
**SEM images of *E. coli *cells before and after treatment by titanium-doped ZnO powders. (a)** Control, **(b)** zinc acetate, **(c)** zinc sulfate, **(d)** zinc nitrate, and **(e)** zinc chloride.

**Figure 7:**
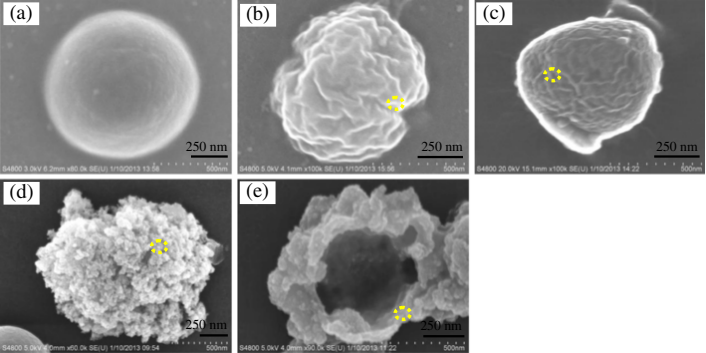
**SEM images of *S. aureus *cells before and after treatment by titanium-doped ZnO powders. (a)** Control, **(b)** zinc acetate, **(c)** zinc sulfate, **(d)** zinc nitrate, and **(e)** zinc chloride.

Figures 6 and 7 show the SEM images of the bacterium before and after treatment with the titanium-doped ZnO powders. In control samples, the *E. coli* cell walls are rough and intact (Figure 6a). However, after being treated with the titanium-doped ZnO powders, the morphologies of *E. coli* cells show changes in varying degrees. Figure 6b,c shows that the *E. coli* cells are damaged slightly after treatment with the ZnO powders prepared from zinc acetate and zinc sulfate. By comparison, the *E. coli* cells are damaged seriously when treated by powders synthesized from zinc nitrate (Figure 6d), and the *E. coli* cells are damaged most seriously being treated by the powders synthesized from zinc chloride (Figure 6e). As shown in Figure 7a, the *S. aureus* cells exhibit well-preserved cell walls. After treatment with titanium-doped ZnO powders synthesized from zinc acetate and zinc sulfate, the crinkling of the *S. aureus* cell walls appeared (Figure 7b,c). However, after being treated with the powders synthesized from zinc nitrate, the *S. aureus* cell walls are damaged into honeycomb (Figure 7d). It is obvious that the effect of the powders synthesized from zinc chloride is the most drastic, and *S. aureus* cells are ruptured (Figure 7e).

From what is mentioned above, we can reach the conclusion that the extent of damage to *E. coli* and *S. aureus* cells is positively related to the antibacterial properties of titanium-doped ZnO powders (Tables 1 and 2). Moreover, many powders are attached to the bacterial cells' surfaces, and the energy-dispersive spectrometer results (Additional file 1) demonstrate that they are titanium-doped ZnO particles (yellow circles in Figures 6 and 7 correspond to the EDS spectra in Additional file 1 in sequence).

### The electrical conductivity of bacterial suspension before and after treatment

**Figure 8:**
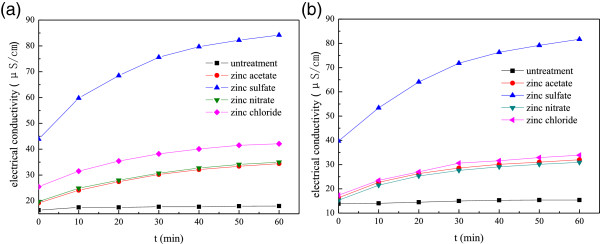
**Electrical conductivity of bacterial suspension before and after treatment by the powders. (a)***E. coli* suspension; **(b)***S. aureus* suspension.

Figure 8 shows the electrical conductance changing trend of the *E. coli* and *S. aureus* suspension treated with titanium-doped ZnO powders synthesized from different zinc salts with different times. The results show that the electrical conductance of the control bacterial suspension is nearly unchanged. However, the electrical conductance of the bacterial suspension increases obviously, which are treated with titanium-doped ZnO powders. In particular, when the bacterial suspension was treated with the powders synthesized from zinc sulfate, its electrical conductivity is much higher than the others no matter on the initial conductivity or on the change of the electrical conductivity. It may be that the powders contain different crystals with the other. It is presumed that bacterial cell wall and cell membrane are damaged by the powders, and the electrolyte is leaked from cells. Furthermore, the electrical conductance increment of bacterial suspension treated by the powders synthesized from zinc chloride is slightly higher than that of zinc acetate and zinc nitrate. This is also related to the antibacterial activities of titanium-doped ZnO powders (Tables 1 and 2).

## Discussion

The bacterial cell wall can provide strength, rigidity, and shape for the cells and can protect the cells from osmotic rupture and mechanical damage. The bacterial cells can be divided into Gram-positive cells and Gram-negative cells according to their cell wall structure. Besides, the wall of Gram-positive cells contains a thick layer of peptidoglycan (PG) of 20 to 80 nm, which is attached to teichoic acids. By contrast, Gram-negative cell walls are more complex, both structurally and chemically. The wall of Gram-negative cell contains a thin PG layer of 2 to 3 nm and an outer membrane of 8 to 10 nm, which covers the surface membrane [37].

**Figure 9:**
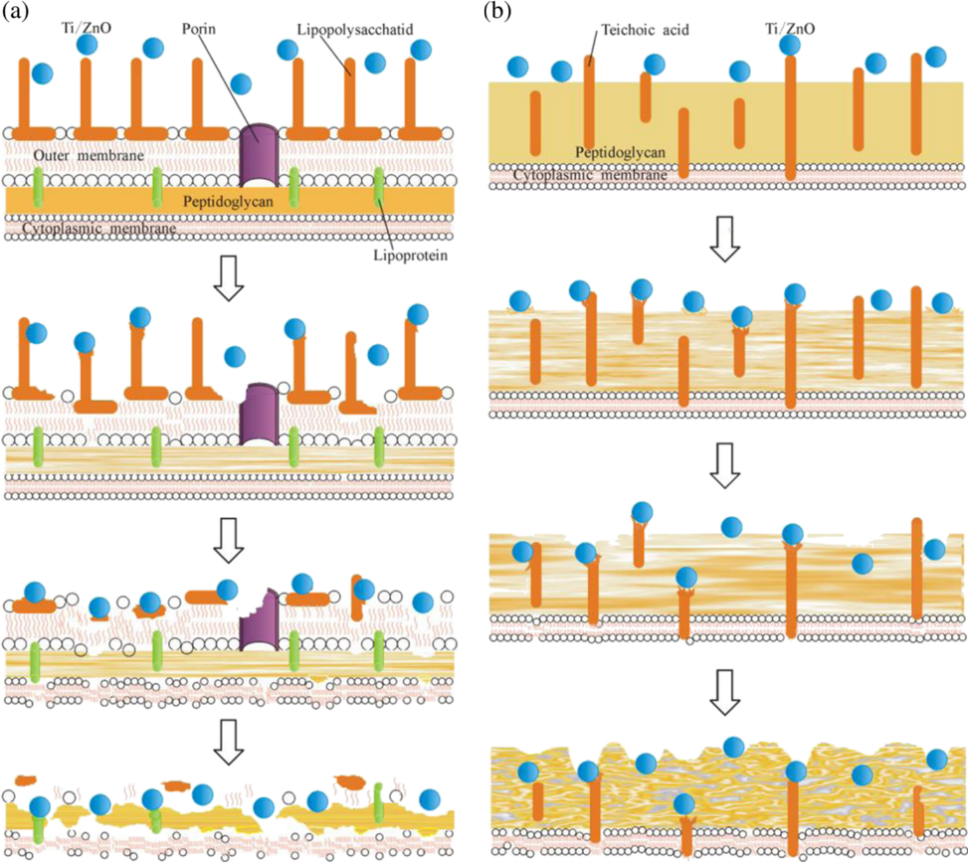
**Antibacterial mechanisms of titanium-doped ZnO powders to (a) *E. coli *** **and (b) *S. aureus.***

In our work, the antibacterial property results show that the titanium-doped ZnO powders against *E. coli* is better than *S. aureus*, the SEM characterizations of the bacterial cells indicate that the powders make the cell wall damage, and the electrical conductance analytic results demonstrate that the electrical conductance added values of *E. coli* suspension are slightly higher than that of *S. aureus* suspension after treatment with the powders. The cell morphologies are affected by the powders' capability of cell wall damage, and the electrical conductance changing values of bacterial suspension are relevant to the damage degree of cell membrane and wall. Moreover, the antibacterial experiments were done in the dark, so there are no active oxide, hydrogen peroxide, and super-oxide. We can conclude that the ZnO powders are attached on the bacterial cell wall through electrostatic interaction, rupturing the cell walls, increasing the permeability, causing the leakage of cytoplasm, and leading to bacterial cell death. Figure 9 schematically illustrates the antibacterial mechanisms of titanium-doped ZnO powders to *E. coli* (Figure 9a) and *S. aureus* (Figure 9b). It may be that the cell walls of *E. coli* are broken easily due to the thin layer of PG, and the cell membranes burst; thus, the antibacterial properties of ZnO powders against *E. coli* is better than *S. aureus*.

Comprehensive analysis reveals that the antibacterial properties of titanium-doped ZnO powders are affected not only by the size but by the crystallinity. When the powders are attached to the bacterial surface, titanium-doped ZnO crystals reacted with PG, teichoic acids, and lipoteichoic acids, and then the structure of bacterial cell wall is damaged. The titanium-doped ZnO powders are crystalline nanorods synthesized from zinc acetate, and its antibacterial activities are lower than the others. Meanwhile, the bacterial cell wall is damaged slightly, and the electrical conductance of bacterial suspension is increased; it indicates that the destroy capacity of the powders to bacterial cell wall and cell membrane is feeblish. This could be because of the weak doping level of titanium in ZnO crystal, although the particle size is smaller than the others. When the titanium-doped ZnO powders are prepared from zinc nitrate, the particles are six prismatic crystals with big size. The bacterial cell wall is damaged seriously, and the electrical conductance of bacterial suspension is increased; it proves that the powders' damage capability to the bacterial cell wall and cell membrane is great. It could be due to good doping level of titanium in ZnO crystal and high dissolving ability of metal ion from the crystals. The titanium-doped ZnO powders are spherical and tooth shape nanoparticles, which are synthesized from zinc chloride. After treatment with them, the bacterial cell wall and cell membrane are damaged seriously, and the increase of electrical conductance of the bacterial suspension is greater than the others. It indicates that the capability of the powders to the cell wall is high and makes the penetrability of cell membrane increased. This is due to high doping level of titanium and small size of particles. When the bacterial suspension is treated by the powders prepared from zinc sulfate, the antibacterial activity is weak and the damage degree of bacterial cell wall is slight. It demonstrates that the antibacterial activities of ZnTiO_3_ and ZnSO_4_ · 3Zn (OH)_2_ crystal are weaker than ZnO.

Furthermore, when the *E. coli* cell walls are damaged by titanium-doped ZnO powders, the holes appeared on the cells; this may be because the thin cell wall and outer membrane are easy to break. When the *S. aureus* cell walls are damaged by the powders, the cell walls become crinkly or honeycomb; this could be due to the thick layer of PG and the PG chemical network structure.

On the basis of the above analysis, it is inferred that the antibacterial properties of the titanium-doped ZnO powders are relevant to the particle size and the crystallinity.

## Conclusions

The titanium-doped ZnO powders with different shapes and sizes were synthesized from different zinc salts. Antibacterial property results show that the titanium-doped ZnO powders have different antimicrobial activities. The antibacterial properties of the powders prepared from zinc chloride are optimal, and its MIC and MBC are lower than 0.25 g L^−1^. Moreover, the antibacterial action of the powders toward *E. coli* is stronger than that towards *S. aureus*.

## Competing interests

The authors declare that they have no competing interests.

## Authors’ contributions

TS guided the thesis writing and experiment. HH wrote the paper and did the experiment. WH and XL did the experiment. SY analyzed the antibacterial mechanism. JL guided the experiment. All authors read and approved the final manuscript.

## Supplementary Material

Additional file 1: Figures S1 and S2 **Figure S1.** EDS of the E. coli cells treated by titanium doped ZnO powders synthetized from different zinc salt (a) zinc acetate; (b) zinc sulfate; (c) zinc nitrate; (d) zinc chloride. **Figure S2.** EDS of the S. aureus cells treated by titanium doped ZnO powders synthetized from different zinc salt (a) zinc acetate; (b) zinc sulfate; (c) zinc nitrate; (d) zinc chloride. Click here for file

## References

[B1] de Moura MR Mattoso LHC Zucolotto V Development of cellulose-based bactericidal nanocomposites containing silver nanoparticles and their use as active food packaging J Food Eng 2012 9 520 524

[B2] Pinto RJ Marques PA Neto CP Trindade T Daina S Sadocco P Antibacterial activity of nanocomposites of silver and bacterial or vegetable cellulosic fibers Acta Biomater 2009 9 2279 2289 19285455 10.1016/j.actbio.2009.02.003

[B3] Priyadarshini S Gopinath V Meera Priyadharsshini N MubarakAli D Velusamy P Synthesis of anisotropic silver nanoparticles using novel strain, *Bacillus flexus* and its biomedical application Colloids Surf, B 2013 9 232 237 10.1016/j.colsurfb.2012.08.018 23018021

[B4] Emamifar A Kadivar M Shahedi M Soleimanian-Zad S Effect of nanocomposite packaging containing Ag and ZnO on inactivation of *Lactobacillus plantarum* in orange juice Food Control 2011 9 408 413

[B5] Hebeish A El-Naggar ME Fouda MMG Ramadan MA Al-Deyab SS El-Rafie MH Highly effective antibacterial textiles containing green synthesized silver nanoparticles Carbohydr Polym 2011 9 936 940

[B6] Tran QT Nguyen VS Hoang TK Nguyen HL Bui TT Nguyen TV Preparation and properties of silver nanoparticles loaded in activated carbon for biological and environmental applications J Hazard Mater 2011 9 1321 1329 21764213 10.1016/j.jhazmat.2011.06.044

[B7] Alarcon EI Udekwu K Skog M Pacioni NL Stamplecoskie KG Gonzalez-Bejar M The biocompatibility and antibacterial properties of collagen-stabilized, photochemically prepared silver nanoparticles Biomater 2012 9 4947 4956 10.1016/j.biomaterials.2012.03.033 22494887

[B8] Young YF Lee HJ Shen YS Tseng SH Lee CY Tai NH Oxicity mechanism of carbon nanotubes on *Escherichia coli* Mater Chem Phys 2012 9 279 286

[B9] Uygun A Kiristi M Oksuz L Manolache S Ulusoy S RF hydrazine plasma modification of chitosan for antibacterial activity and nanofiber applications Carbohydr Res 2011 9 259 265 21159329 10.1016/j.carres.2010.11.020

[B10] Selvam S Rajiv Gandhi R Suresh J Gowri S Ravikumar S Sundrarajan M Antibacterial effect of novel synthesized sulfated beta-cyclodextrin crosslinked cotton fabric and its improved antibacterial activities with ZnO, TiO_2_ and Ag nanoparticles coating Int J Pharm 2012 9 366 374 22627018 10.1016/j.ijpharm.2012.04.069

[B11] Necula BS van Leeuwen JP Fratila-Apachitei LE Zaat SA Apachitei I Duszczyk J In vitro cytotoxicity evaluation of porous TiO_2_-Ag antibacterial coatings for human fetal osteoblasts Acta Biomater 2012 9 4191 4197 22813846 10.1016/j.actbio.2012.07.005

[B12] Dallas P Sharma VK Zboril R Silver polymeric nanocomposites as advanced antimicrobial agents: classification, synthetic paths, applications, and perspectives Adv Colloid Interface Sci 2011 9 119 135 21683320 10.1016/j.cis.2011.05.008

[B13] Carmen Steluta C Simona Liliana I Le Phillippe C Liliana Violeta C Daniela P Antibacterial activity of silver-doped hydroxyapatite nanoparticles against gram-positive and gram-negative bacteria Nanoscale Res Lett 2012 9 324 332 10.1186/1556-276X-7-324 PMC3422172 22721352

[B14] Hwang JJ Ma TW Preparation, morphology, and antibacterial properties of polyacrylonitrile montmorillonite/silver nanocomposites Mater Chem Phys 2012 9 613 623

[B15] Thangaraju N Venkatalakshmi RP Chinnasamy A Kannaiyan P Synthesis of silver nanoparticles and the antibacterial and anticancer activities of the crude extract of *Sargassum polycystum* C Agardh Nano Biomed Eng 2012 9 2 89 94

[B16] Tripathi RM Rana D Shrivastav A Singh RP Shrivastavd BR Biogenic synthesis of silver nanoparticles using *Saraca indica* leaf extract and evaluation of their antibacterial activity Nano Biomed Eng 2013 9 1 50 56

[B17] Prucek R Tucek J Kilianova M Panacek A Kvitek L Filip J The targeted antibacterial and antifungal properties of magnetic nanocomposite of iron oxide and silver nanoparticles Biomater 2011 9 4704 4713 10.1016/j.biomaterials.2011.03.039 21507482

[B18] Trujillo NA Oldinski RA Ma H Bryers JD Williams JD Popat KC Antibacterial effects of silver-doped hydroxyapatite thin films sputter deposited on titanium Mater Sci Eng C 2012 9 2135 2144

[B19] Chan YH Huang CF Ou KL Peng PW Mechanical properties and antibacterial activity of copper doped diamond-like carbon films Surf Coat Technol 2011 9 1037 1040

[B20] Pramanik A Laha D Bhattacharya D Pramanik P Karmakar P A novel study of antibacterial activity of copper iodide nanoparticle mediated by DNA and membrane damage Colloids Surf, B 2012 9 50 55 10.1016/j.colsurfb.2012.03.021 22521682

[B21] Osamu Y Toshiaki O Kelly A Fukuda M Antibacterial characteristics of CaCO_3_–MgO composites Mater Sci Eng B 2010 9 208 211

[B22] Dong CX Song DL John C Orville Lee M He GH Deng YL Antibacterial study of Mg (OH)_2_ nanoplatelets Mater Res Bull 2011 9 576 582

[B23] Trandafilović LV Božanić DK Dimitrijević-Branković S Luyt AS Djoković V Fabrication and antibacterial properties of ZnO–alginate nanocomposites Carbohydr Polym 2012 9 263 269

[B24] Hoda N Topel O Budama L BA C Synthesis of ZnO nanoparticles using PS-b-PAA reverse micelle cores for UV protective, self-cleaning and antibacterial textile applications Colloids Surf, A 2012 9 132 139

[B25] Martins NCT Freire CSR Neto CP Silvestre AJD Causio J Baldi G Antibacterial paper based on composite coatings of nanofibrillated cellulose and ZnO Colloids Surf, A 2013 9 111 119

[B26] Jalal R Goharshadi EK Abareshi M Moosavi M Yousefi A Nancarrow P ZnO nanofluids: green synthesis, characterization, and antibacterial activity Mater Chem Phys 2010 9 198 201

[B27] Abou-Okeil A El Shafei A ZnO/carboxymethyl chitosan bionano-composite to impart antibacterial and UV protection for cotton fabric Carbohydr Polym 2011 9 920 925

[B28] Anitha S Brabu B Thiruvadigal DJ Gopalakrishnan C Natarajan TS Optical, bactericidal and water repellent properties of electrospun nano-composite membranes of cellulose acetate and ZnO Carbohydr Polym 2012 9 1065 1072 10.1016/j.carbpol.2013.05.003 24066357

[B29] Karunakaran C Rajeswari V Gomathisankar P Optical, electrical, photocatalytic, and bactericidal properties of microwave synthesized nanocrystalline Ag–ZnO and ZnO Solid State Sci 2011 9 923 928

[B30] Thangavelu Kavitha AIG Lee KP Park SY Glucose sensing, photocatalytic and antibacterial properties of graphene–ZnO nanoparticle hybrids Carbon 2012 9 2994 3000

[B31] Nair MG Nirmala M Rekha K Anukaliani A Structural, optical, photo catalytic and antibacterial activity of ZnO and Co doped ZnO nanoparticles Mater Lett 2011 9 1797 1800

[B32] Talebian N Nilforoushan MR Zargar EB Enhanced antibacterial performance of hybrid semiconductor nanomaterials: ZnO/SnO_2_ nanocomposite thin films Appl Surf Sci 2011 9 547 555

[B33] Phan DT Chung GS Effects of defects in Ga-doped ZnO nanorods formed by a hydrothermal method on CO sensing properties Sens Actuators, B 2013 9 191 197

[B34] Li Q Chen Y Luo L Wang L Yu Y Zhai L Photoluminescence and wetting behavior of ZnO nanoparticles/nanorods array synthesized by thermal evaporation J Alloys Compd 2013 9 156 160

[B35] Lin Y Yang Z Cheng J Preparation, characterization and antibacterial property of cerium substituted hydroxyapatite nanoparticles J Rare Earths 2007 9 452 456

[B36] Selvam S Sundrarajan M Functionalization of cotton fabric with PVP/ZnO nanoparticles for improved reactive dyeability and antibacterial activity Carbohydr Polym 2012 9 1419 1424

[B37] Hajipour MJ Fromm KM Ashkarran AA Jimenez de Aberasturi D de Larramendi IR Rojo T Antibacterial properties of nanoparticles Trends Biotechnol 2012 9 499 511 22884769 10.1016/j.tibtech.2012.06.004

