# Supplementary material for: Preparation and antibacterial properties of titanium-doped ZnO from different zinc salts
Source: Nanoscale Res Lett. 2014 Feb 27;9(1):98. doi: 10.1186/1556-276X-9-98 (PMC4015756; doi:10.1186/1556-276X-9-98)
Supplement: Additional file 1: Figures S1 and S2 — Figure S1. EDS of the E. coli cells treated by titanium doped ZnO powders synthetized from different zinc salt (a) zinc acetate; (b) zinc sulfate; (c) zinc nitrate; (d) zinc chloride. Figure S2. EDS of the S. aureus cells treated by titanium doped ZnO powders synthetized from different zinc salt (a) zinc acetate; (b) zinc sulfate; (c) zinc nitrate; (d) zinc chloride. [file 1556-276X-9-98-S1.doc]

(a)

(b)

(c)

(d)

Fig.S1 EDS of the *E. coli* cells treated by [titanium](app:ds:titanium) doped ZnO powders [synthetize](app:ds:synthetize)d from different [zinc](app:ds:zinc) [salt](app:ds:salt)

(a) zinc acetate; (b) zinc sulfate; (c) zinc nitrate; (d) zinc chloride

(a)

(b)

(c)

(d)

Fig.S2 EDS of the *S. aureus* cells treated by [titanium](app:ds:titanium) doped ZnO powders [synthetize](app:ds:synthetize)d from different [zinc](app:ds:zinc) [salt](app:ds:salt)

(a) zinc acetate; (b) zinc sulfate; (c) zinc nitrate; (d) zinc chloride
